# Metabolomics and EMT Markers of Breast Cancer: A Crosstalk and Future Perspective

**DOI:** 10.3390/pathophysiology29020017

**Published:** 2022-05-27

**Authors:** Ajay Kumar Pal, Prateek Sharma, Alishan Zia, Deepali Siwan, Dipali Nandave, Mukesh Nandave, Rupesh K. Gautam

**Affiliations:** 1 Department of Pharmacology, Delhi Pharmaceutical Sciences and Research University, New Delhi 110017, India; ajaypal7894@gmail.com (A.K.P.); prateek2009@gmail.com (P.S.); alishanzia2828@gmail.com (A.Z.); deepalisiwan6@gmail.com (D.S.); 2 Department of Dravyaguna, Karmavir V. T. Randhir Ayurved College, Boradi 425428, India; dipalinandave@gmail.com; 3 Department of Pharmacology, MM School of Pharmacy, Maharishi Markandeshwar University, Ambala 134007, India

**Keywords:** breast cancer stem cells (BCSCs), metastasis, EMT-transcription factors, triple-negative breast cancer (TNBC), tumor microenvironment, chemoresistance

## Abstract

Cancer cells undergo transient EMT and MET phenomena or vice versa, along with the parallel interplay of various markers, often correlated as the determining factor in decoding metabolic profiling of breast cancers. Moreover, various cancer signaling pathways and metabolic changes occurring in breast cancer cells modulate the expression of such markers to varying extents. The existing research completed so far considers the expression of such markers as determinants regulating the invasiveness and survival of breast cancer cells. Therefore, this manuscript is crosstalk among the expression levels of such markers and their correlation in regulating the aggressiveness and invasiveness of breast cancer. We also attempted to cover the possible EMT-based metabolic targets to retard migration and invasion of breast cancer.

## 1. Introduction

Breast cancer is the most conspicuous health concern among women worldwide with a mortality rate of 6.9%, and accounting for 11.7% of all cancer-related cases, as per reports obtained from the GLOBOCAN survey conducted in the year 2020 [1]. Despite the advancement of cancer research and technologies, defining breast cancer is becoming more troublesome because it is not just a disease but rather a heterogeneous disease that shows diverse histopathological highlights, hereditary and genomic variability, and discrete prognostic results [2,3]. More than 90% of cancer-related deaths have been associated with metastasis of primary tumors at distant sites due to migration and invasion of breast cancer cells. Of them, the epithelium-originated malignant tumors are approximately above 80% [4,5]. Besides the advancements in the personalized treatment regimens for breast cancer subtypes, there is also simultaneous progress in metastasis of breast cancer to the distant organs/tissues (brain, bone, lung, liver, and lymph node), a giant factor behind the rising mortality of breast cancer patients [6]. Also, the developing unresponsiveness to chemotherapeutics (drug resistance), tumor relapse, poor survival, immune suppression, and advanced metabolic rewiring are the challenges prominently associated with the consequences of metastatic breast cancer. The pathophysiology of metastasis is regulated by the EMT and MET programs. Thus, breast cancer metastasis has led us to study both the EMT and MET programs, their markers, and the associated metabolic inhibitors and adjuvant therapeutics blocking them. Therefore, the review focuses on expanding the potential of epigenetic correlation of breast cancer with metabolomics of the EMT program and markers associated with each subtype. This review could open new doors or avenues for designing novel adjuvant therapeutics for metastatic breast cancer.

## 2. Status of Metastasis with Breast Cancer Subtypes, Metabolic Rewiring, EMT and MET—An Insight

### 2.1. Breast Cancer Subtypes

On-premises of immuno-histochemical articulation of receptors, specifically estrogen receptor (ER), progesterone receptor (PR), human epidermal development receptor 2 (HER2), breast cancer can be partitioned into four significant subcategories, ER^+^/PR^+^/HER2^−^, ER^+^/PR^+^/HER2^+^, ER^−^/PR^−^/HER2^+^, and ER^−^/PR^−^/HER2^−^. Based on intrinsic gene expression profiles, breast cancers can be divided into of four subtypes: Luminal A, Luminal B, HER2^+^, and basal-like, each of which is displayed to have various visualizations [3,7,8,9]. The latest molecular classification of breast cancer can be incorporated into six subtypes of malignant growth of the breast. These are ordinary-like (with expression profile as non-cancer breast tissue); luminal A and B (mostly estrogen receptor ER^+^ cancers, with an expression of epithelial markers; luminal B exhibit high Ki67 index and more terrible anticipation contrasted with luminal A); HER2^+^ (high expression of oncogene- ERBB2); basal-like (expressing basal cytokeratin and different markers normal for the myoepithelium of the typical mammary organ); and claudin-low [enhanced in epithelial-to-mesenchymal transition (EMT) highlights, immune system responses, and stem-cell related natural cycles]. Claudin-low and basal-like subtypes have a place with the gathering of triple-negative breast cancer (TNBC), which are characterized by the absence of progesterone receptor (PR), ER, and HER2 articulations, and the presence of androgen receptor (AR) therefore known as LAR breast cancer subtypes with a high recurrence of visceral metastases [10]. The luminal A, luminal B, and HER2-enriched breast cancer cells hold most epithelial features, whereas basal-like tumors show both basal and mesenchymal features [11,12]. The basal-like breast cancer cells are constitutively more intrusive. Further, HER2- enriched growths foster metastatic illness. Although the counter HER2 (Herceptin, otherwise called trastuzumab) therapy has been effectively used to treat metastatic HER-2 breast malignant growth, again, bought resistance is a significant issue with trastuzumab treatment [13,14,15]. A high proportion of HER2-over-expressing breast malignant growth patients who experience protection\resistance from trastuzumab progress to foster brain metastases. The two-year endurance rate for cerebrum metastasis is under 2% [16,17]. The diverse molecular stratification of breast cancer based on epigenetics and details about the site of metastasis have also been given in Table 1.

### 2.2. Metabolic Rewiring, EMT, MET and Their Impact on Breast Cancer Progression

The primary breast cancer tumor cells continuously use supplied nutrients, oxygen, energy, and metabolites from the surrounding parenchymal breast cells to multiply and grow uncontrollably. With the advancing uncontrolled multiplication of cancer cells, there is a tremendous burden on the primary tumor cells to fulfill their unmet demands. Thus, to meet their energy and nutrient demands, they reprogram or rewire their metabolism to direct and promote multistep metastasis, proliferation, and survival. Such a process is known as metabolic reprogramming or metabolic rewiring. The metabolites recruited by primary cancer cells influence the metastatic cascade, induction of EMT, survival of cancer cells in circulation, and metastatic colonization at distant sites [20]. Thus, metabolic reprogramming is an emerging hallmark of cancer, which is observed in breast cancer too. Breast cancer cells rewire their cellular metabolism to meet the demands of survival, proliferation, and metastasis.

**Figure 1:**
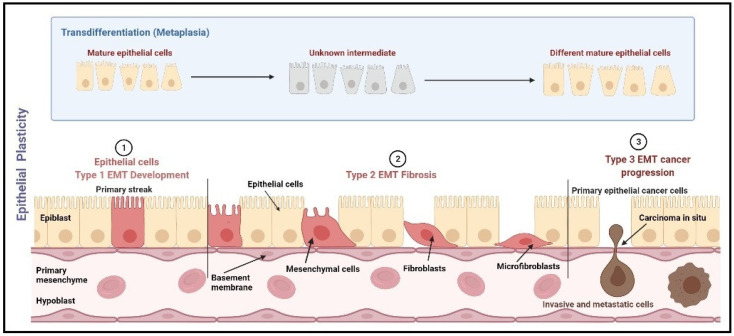
EMT categorization based on epithelial plasticity. EMT is categorized into three types, namely mesenchymal, fibroblast, and metastatic. Type 1 EMT occurs during the mesenchymal transformation of primary epithelia, which occurs during gastrulation, neural crest cell initiation from neuroepithelial cells, and production of endocardial cells (cushion tissues) from cardiac endothelial cells. Type 2 EMT includes the transition of secondary epithelial cells into the fibroblast tissues, as observed in the wound healing process, tissue regeneration, and fibrosis in adult tissues. Type 3 EMT occurs mostly in carcinoma cells which transit from epithelial cells to mesenchymal cells leading to the formation of metastatic tumor tissue [27]. Image created using Biorender (https://biorender.com/, accessed date 15 January 2022).

Not only this, but the primary breast cancer tumor cells also carry a subpopulation of stem cells known as BCSCs, which renew themselves and regenerate into a new tumor which carries potential to metastasis [21]. The primary tumors with subsequent systemic treatments (hormone therapy, chemotherapy, or targeted therapy) are enriched with BCSCs, which carry signature genes to mediate the process of EMT [16,17,22,23]. The process of EMT is a dynamic biological process characterized by a reversible transition in cell state, i.e., transdifferentiation of immotile epithelial cells to motile mesenchymal cells [24,25,26]. A descriptive picture of EMT categorization based on epithelial plasticity is shown in Figure 1.

The EMT process starts with the polarization of epithelial cells and loosening of the tight junction with low expression of epithelial markers (α-catenin, E-cadherin, and γ-catenin) to acquire mesenchymal properties, i.e., high motility, invasiveness, and stipulated resistance to programmed cell death (apoptosis), carrying a high expression of mesenchymal markers, fibronectin, N-cadherin, and vimentin (CDH2) [28]. Collectively, the whole sketch is regulated by the precise interplay between signaling pathways, transcription factors (TFs)– Twist-linked protein (Twist), Snail (SNAI1), Slug (SNAI2), zinc finger E-box-binding homeobox (ZEB1), microRNAs, and therapeutics as well [29]. Epigenetic regulation has also been implicated in diversifying the EMT/MET transdifferentiation. The induction of EMT is also dependent on certain endogenous and exogenous moieties, which might exaggerate or suppress the expression of markers regulating EMT and thus play a crucial role in influencing the invasiveness of breast cancer cells [30].

Metastasis of cancer cells initiates with the EMT, undergoing primary tumor cells carrying highly expressed EMT markers (Vimentin, N-cadherin, BCSCs), mediating the loss of cellular adhesion. Such a loss of cellular adhesion promotes local migration, invasion, and intravasation (entry into blood vessels), which survive as single cells or get coated with platelets and disseminate to the existing vessels, i.e., extravasation into the parenchyma of distant organs and tissues. The colonization of EMT and partial EMT cells then undergo re-differentiation into epithelial phenotype, termed mesenchymal-to-epithelial transition (MET), through interaction with the tumor microenvironment. The cancer cells undergoing the MET program are characterized by local expression of MET markers such as E-cadherin, occluding, and cytokeratin [27].

The EMT program is also essential for various physiological and pathological developments of body tissues and organs. It has been documented that EMT is an essential component governing physiological control during the development of the embryo [26]; primitive mesenchymal cell types are an essential part of the mesoderm and endoderm [26]. In addition, partial and reversible EMT is observed during the morphogenesis of mammary glands [31]. The progenitor breast or breast stem cells of terminal buds of the breast begin to elongate and migrate during puberty, leading to branching morphogenesis [32]. These epithelial cells acquire mesenchymal traits such as loss of apical-base polarity [33,34] and increased expression of the transcription factors SNAI1 and Twist in a transitory manner [35,36]. Extracellular substances cause EMT, such as epidermal growth factor (EGF) and hepatocyte growth factor (HGF), which influence cells in terminal buds. Overexpression of HGF produces hyperplastic branched morphogenesis in the mouse mammary gland, whereas inhibition of HGF signaling prevents lateral branch budding [37]. Because both epithelial and mesenchymal lineages are required for optimal mammary gland function, branched morphogenesis is a highly flexible process with an incomplete EMT program. ELF5 and OVOL2, the transcription factors that prevent EMT in terminal buds, have recently been identified as the guardians of mammary epithelial development [38]. During pregnancy and breastfeeding, ElF5 is the key regulator for transforming of luminal progenitor cells into alveolar cells [39,40]. As a result, the partial EMT state, partial earning of mesenchymal traits, and the preservation of some epithelial properties are crucial throughout mammary gland development.

Besides the function of EMT in the development of tissues, it plays a prime role in the development of various cancers. It is documented that approximately 80% of cancer-related deaths in humans are caused by cancers of epithelial tissue i.e., breast, colon, kidney, liver, lung, pancreas, prostate, and ovary [41]. The progression of the EMT program leads to metastatic stages, i.e., invasion and migration, which worsen the management, and thus patients become fatal [42].

The EMT program highly resembles the claudin and basal subtypes of breast cancer more than the luminal A/B subtype [43]. Also, the genesis of the tumor and its progression are positively linked with the attainment of mesenchymal features. Thus, claudin-low and basal subtypes of breast cancer are found to be more aggressive and proliferative. The downregulation of EMT potentiators, namely, Snail, TWIST, and ZEB, in breast cancer cells of both humans and mice significantly inhibits metastasis induced through mammary fat pad or tail vein injection [43,44,45,46,47,48]. For instance, the depletion of Snail in MMTV-PYMT mice completely treated 95% of lung metastasis [49]. Such findings can be very well correlated with a significant increase in the rate of metastasis upon activation of EMT in human breast cancer cells [46]. Thus, it is evident that EMT is a pivotal component of metastatic events.

The incompetence of metastatic events to handle an ectopic environment for survival necessitates the underlying mechanism to explore MET program too. The transition or the reversal of mesenchymal cancer cells to epithelial cancer cells leads to secondary tumor formation with new metabolically different phenotypes of breast cancer and the birth of late metastatic colonies. Such reversal of initial EMT at the primary tumor site is termed the mesenchymal-epithelial transition (MET), in which mesenchymal cancer cells transform into epithelial cancer cells, leading to secondary tumor formation with new metabolically different phenotypes of metastatic breast cancer cells at secondary sites via colonization of disseminated tumor cells (DTCs) [50,51,52,53]. The paradigm of early metastasis is featured in a small proportion of primary tumor cells containing high CD44 and low CD24 stem cell-like features which carry the potential to leave the primary tumor early to metastasize at distant sites. Recently, this well-accepted concept of “late metastasis” has been challenged by some groups who have shown that tumor cell dissemination occurs early in the establishment of the primary tumor [49,54]. The paradigm of early metastasis with a consistent proportion of primary tumor cells enriched with high CD44, and low CD24 (high CD44/low CD24) stem cell-like features carries the potential to migrate primary tumor sites and form metastatic colonies in distant sites. Hence, it is evident that high CD44/low CD24 phenotypes of breast cancer cells are highly associated with EMT features of BCSCs with drastic malignant features [12,55]. However, the disseminated cells appear to have a mesenchymal phenotype, which is inconsistent with the finding of epithelial-type breast cancer nodules in ectopic tissues.

The evidence that elevated levels of epithelial microRNA family (miR)-200 in primary breast cancers led to successful metastasis [56] was rather surprising. MiR-200 is a critical regulator of the EMT phenotype of breast cancer, which promotes the re-expression of E-cadherin through repression of transcription factor genes- ZEB to implicate invasion and metastasis [57,58,59,60,61]. Indeed, certain MiRs also interplay in metastatic colonization, supporting the MET role. However, in bladder and prostate cancer systems, metastatic colonization was linked with epithelial cell lines of these cancers rather than mesenchymal ones, as well as high expression of self-renewal genes and pluripotency [62]. In another study, such overexpression of these genes was abolished upon induction of EMT with reduced tumorigenicity and abolished metastatic potential. Such studies illustrate the pro-metastatic role of EMT.

EMT confers greater tumor-initiating and metastatic ability in breast cancer cells [63,64]. As previously described, the EMT process is characterized by elevated expression of mesenchymal markers (N-cadherin, Vimentin) and diminished expression of epithelial markers (E-cadherin) [65]. Multiple EMT-TFs, including Twist, Snail, Slug, ZEB, and other regulatory molecules, including transforming growth factor-β (TGF-β), platelet-derived growth factor, HGF, and EGF drive the EMT process [47,66]. Mechanically, these TF-EMTs also trigger changes in gene expression and signaling cascades implicated in the stem, invasion, and metastasis. Thus, inhibiting the activation of the described EMT-TFs is critical for restraining breast cancer invasion and its metastatic potential.

It is well established that EMT equips breast cancer cells with mesenchymal characteristics, resulting in greater resistance to different therapy modalities. EMT confers breast cancer cells with similar features to BCSC, such as developing resistance to therapies, which may be connected with the control of cell-specific genes (for survival, stem cell maintenance, and resistance to therapy) [55,67,68]. For instance, breast cancer mesenchymal cells exhibit high resistance to the antitumor immune response. Immunotherapy was less effective against breast mesenchymal cancer cells than against matching epithelial tumors. Mesenchymal cells are defined by a high level of PD-L1 expression and a low level of MHC-I expression [69]. EMT also impairs breast cancer cells’ vulnerability to T-cell-mediated immune surveillance. It is also responsible for drug resistance in breast cancer cells [70,71]. It was evident from the findings that cyclophosphamide therapy resistance was observed in breast cancer cells undergoing EMT with due tolerance to apoptosis and high expression of chemoresistance genes [67]. Therefore, EMT molds both metastasis and therapy resistance, indicating the progression of breast cancer.

Ample reports are available detailing the mutual association of EMT with metabolic reprogramming in cancer cells [72]. To meet their unmet energy requirements, cancer cells alter their metabolic phenotype to recruit ATP and other important metabolic intermediates for sustaining survival, proliferation, and metastasis [73]. EMT is associated with extensive metabolic rewiring in breast cancer cells to meet their energy demands for enhanced motility and invasion in hypoxic and nutrient-depleted environments, but only as regulated by EMT. Still, the process of metabolic adaptation is only partially understood [72,74]. Shaul et al. found multiple mesenchymal metabolic signature genes by analyzing the expression of metabolic genes in many tumor cell lines expressing mesenchymal characteristics. These genes were discovered to be up-regulated in epithelial cells of the human breast following EMT induction. This discovery suggests that the EMT program may have a direct effect on metabolic gene expression [75]. Additional research is being conducted to determine how uncontrolled metabolic pathways influence the beginning and progression of TMS. This section aims to discuss how metabolic pathways activate TF-TMS and trigger the TMS process, which contributes to breast cancer growth. The characteristics and expression of different EMT markers in breast cancer are shown in Table 2.

## 3. Metabolomics of Breast Cancer in Driving the EMT Marker Expression and Their Blockers

Metabolomics (the study of metabolomes) can be carried out via targeted and untargeted approaches. The targeted approach of metabolomics focuses on identifying the metabolites of interest or a pathway with the basis of that particular pathway or metabolite being in the metabolome composition of an investigated sample. The untargeted approach of metabolomics focuses on identifying and quantifying the metabolites in a biological sample. Nuclear magnetic resonance (NMR) and mass spectroscopy (MS) are the prime tools for metabolome analysis. For precise and advanced estimation of the metabolome in a biological sample, hyphenation of separation techniques can be coupled likewise; LC-MS or GC-MS and LC-NMR or GC-NMR [76]. The obtained results from these tools provide broad insight into pathological mechanics from isolated biomarkers. However, such results can be measured and interpreted using computational models to clinically correlate with EMT/MET markers, which will lead us to specifically diagnose breast cancer subtypes and can be hypothesized for targeted mechanistic therapeutics [77,78,79]. The interpreted findings provide early disease diagnosis, toxicity analysis, nutritional status, the action of the drug, and associated resistance to chemotherapy [80]. Our review discusses the relevance of metabolomics of breast cancer and EMT profiling as a basis to mediate cancer stemness, chemoresistance, migration, and invasion.

The EMT program has abruptly influenced the metabolism of amino acids and glucose in breast tumor cells, as documented by various reports. In addition, such metabolism can be dysregulated or inhibited using various endo-exogenous agents. Breast cancers have a metabolic profile distinct from normal mammary epithelial cells, and drug-sensitive breast cancers have a metabolic profile distinct from resistant breast cancer cells. As a result, metabolic pathway analysis enables a better understanding of the metabolic abnormalities, resulting in more invasive and metastatic cancers [81]. It has been detailed below how metabolomics plays a pivotal role in regulating the EMT program through various metabolic targets/enzymes in glucose, lipid, and amino acid metabolism. Thus, we have summarized key metabolites’ roles in regulating the EMT program and their inhibitors to retard the EMT program of breast cancer, as shown in Table 3.

### 3.1. Glucose Metabolism

Normal human cells use glucose as an energy source in the presence of oxygen. In the cytosol, glucose is digested to make pyruvate, which enters the mitochondria and is oxidized in the Krebs cycle to release ATP as the primary source of cellular energy storage. Even under aerobic conditions, however, the majority of pyruvate produced by cancer cells is diverted from the mitochondria and transformed into lactate through lactate dehydrogenase. This effect is most frequently noticed in low-oxygen environments. Aerobic glycolysis, or the “Warburg effect,” is a mechanism that results in the production of lactate in the presence of oxygen [82,83,84,85]. Breast cancer cells have increased glucose uptake [82] associated with activated oncogenes (RAS and MYC) and mutant tumor suppressors (TP53), which interfere with proliferation, suppression of growth, and significant apoptosis. During neoplastic growth, progressive hypoxia occurs due to inefficient neovascularization, leading to the expression of multiple enzymes involved in the glycolytic pathway [86]. In addition to providing energy and biomolecules to cancer cells, the glycolytic diversion also contributes to intracellular signaling, thus validating a symbiotic linkage between the tumor microenvironment with tumor cells and the adjacent stroma. The lactate acts as a source of energy and molecular signaling, mimicking the high aerobic physiological mechanisms in cancer cells. However, the complex tumor microenvironment and its interconnections between different cell types make it difficult to understand the lactate circuit [87].

Cancer cells are defined by a metabolic switch from mitochondrial oxidative metabolism to aerobic glycolysis to rapidly generate sufficient energy and critical intermediates, which are required to increase their invasive and metastatic potential [88]. EMT is associated with enhanced aerobic glycolysis and the upregulation of glycolysis-related enzymes in breast cancer [89]. For instance, pyruvate kinase M2 (PKM2), involved in the limiting step of glycolysis, is strongly related to the EMT process. Fructose 1,6-bisphosphatase (FBP1) inhibits PKM2 activation, inhibiting glycolysis, while simultaneously boosting the activity of mitochondrial complex I, thereby enhancing oxidative phosphorylation (OXPHOS). As a result, FBP1 deficiency is required to boost glycolytic intermediates for biosynthesis and promote ATP generation, resulting in enhanced BCSC-like features for snail-mediated EMT [90]. Microenvironmental factors can also trigger EMT by altering PKM2 expression and reprogramming the glycolytic phenotype of breast cancer. Leptin, an adipokine, is involved in the prognosis of breast cancer and promotes EMT via high PKM2 expression and activation of the PI3K/AKT signaling cascade [91]. In addition, it was explored that phosphorylating PKM2 promotes similar cellular characteristics to BCSC via activating signaling downstream of the self-associated protein (YAP). PKM2 is phosphorylated on tyrosine 105 by activated kinases, which confers on PKM2 an oncogenic activity in breast cancer cells by increasing YAP nuclear translocation. Silencing YAP impairs the BCSC characteristics mediated by oncogenic kinases, hence inhibiting EMT and reversing chemotherapy resistance [92,93]. Taken together, our findings indicate that PKM2 and its downstream signaling may be viable targets for reversing the mesenchymal phenotype.

Pyruvate dehydrogenase kinase 1 (PDK1) is another critical glycolytic enzyme that prevents the pyruvate dehydrogenase complex from initiating the tricarboxylic acid (TCA) cycle. By increasing glycolytic metabolism, PDK1 recruitment has been associated with liver metastases. Additionally, PDK1 is necessary for EMT induction. Inhibiting PDK1 efficiently decreases mesenchymal markers and prevents lung-specific metastasis [94]. Exogenous expression of PDK1 enables PDK1 to silence breast cancer cells, allowing them to revert to a mesenchymal state. Additionally, a long noncoding RNA called H19 is required for glycolytic activity and BCSC characteristics. It is highly related to PDK1 expression [95]. Silencing H19 abolishes PDK1 expression under hypoxia, glycolysis, and self-renewal circumstances. Notably, aspirin has been shown to significantly reduce the characteristics of BCSC by inhibiting both H19 and PDK1, which provides more information for blocking the EMT process [96].

Phosphoglucose isomerase (PGI) influences EMT at the first stage of cancer metastasis and MET during the final stage of metastasis during colonization cancer through catalyzing the interconversion of glucose 6-phosphate and fructose-6-phosphate. PGI/AMF overexpression, in particular, causes EMT in normal mammary epithelial cells, allowing them to escape the initial tumor. Additionally, inhibiting PGI/AMF expression promotes MET in aggressive breast cancer cells, facilitating their colonization and development in secondary locations [97]. A subsequent study discovered that overexpression of PGI/AMF enhances EMT via increasing the DNA-binding activity of nuclear factor-kB (NF-kB) and further regulating ZEB transcription. MicroRNA-200 can inhibit ZEB expression, implying that miR-200s may be a therapeutic target for reversing PGI/AMF-induced EMT [98].

Cancer cells can undergo the metabolic switch from OXPHOS to glycolysis in hypoxia during rapid proliferation [99]. Therefore, decreased OXPHOS activity has been commonly described in breast cancer cells. Decreased OXPHOS activity may be the result of a mutation in mitochondrial DNA (mtDNA) or lower mtDNA content, which encodes OXPHOS protein complexes [100]. The reduced mtDNA content promotes a calcineurin-dependent retrograde mitochondrial signaling pathway, which induces the EMT process and BCSC properties [101]. The role of reduced mtDNA content and reduced OXPHOS activity in EMT induction could provide new targets for metastasis.

Additionally, oxidative stress plays a critical role in the induction of EMT. A new hypothesis is that reducing reactive oxygen species (ROS) produced by mitochondria can initiate the EMT process. While both BCSC states exhibit increased expression of glycolysis-related genes, mesenchymal and epithelial BCSCs respond to oxidative stress via unique metabolic pathways and redox potentials. In this setting, increased ROS induces the epithelialization of mesenchymal BCSCs. As a result, mesenchymal BCSCs exhibit a decreased OXPHOS potential and a low ROS level [102]. NADH and NADPH are significant sources of reducing equivalent ROS detoxification involvement and hence serve as critical contributors in decreasing intracellular ROS [103]. As a result, the NAD (P) H level can act as a link between ROS and the EMT process. Overexpression of NAD (P) H: quinone oxidoreductase-1 (NQO1) promotes pyruvate kinase expression in the liver and red blood cells (PKLR) in breast cancer. NQO1 interacts with PKLR to promote glycolysis while preserving NAD (P) H homeostasis. By silencing NQO1, intracellular ROS levels are significantly increased, which may hinder the EMT process [104]. As a result, the NQO1/PKLR network promotes EMT induction and may be a useful therapeutic target for inhibiting EMT. Consistently, it has been demonstrated that C-terminal binding protein (CtBP), a critical epigenetic effector downstream of increased NAD (P) H, induces mesenchymal and BCSC features in breast cancer cells [105]. CtBP inhibition dramatically inhibits the EMT process, establishing CtBP as a therapeutic target for reversing EMT. However, there is disagreement about whether higher ROS levels in breast cancer may potentially be associated with EMT and BCSC-like features [106,107]. Matrix metalloproteinase-3 overexpression, a signal from the breast cancer microenvironment, raises the quantity of reactive oxygen species (ROS) in breast cancer cells. Increased ROS expression further promotes snail and EMT expression [108]. Consistently, increased mitochondrial OXPHOS and ROS levels have been implicated in the maintenance of BCSCs in TNBCs [109]. Additional research is required to elucidate the precise role of ROS in promoting EMT in breast cancer.

### 3.2. Lipid Metabolism

In addition to glucose metabolism, abnormal lipid metabolism plays a role in the expansion of EMT in breast cancer. According to earlier research, de novo lipogenesis is enhanced by oncogenic signaling in breast tumor cells, allowing for the creation of sufficient membrane phospholipids and signal molecules in preparation for invasion and metastasis. Sterol regulatory element-binding transcription protein 1 (SREBP1), the main transcriptional promoter of lipogenesis, can increase de novo lipogenesis by increasing the expression of key lipogenesis. SREBP1 inhibits the expression of E-cadherin in breast cancer by forming a co-repressor structure with Snail and histone deacetylase. SREBP1 inhibition may be mediated by miR-18a-5p, which inhibits EMT and breast cancer lung metastasis [110]. In addition, the lipogenic enzyme fatty acid synthase (FASN) is required for EMT expansion in breast tumor. Cerulenin, a FASN blocker, has the ability to slow down the EMT process dramatically [111]. Moreover, FASN inhibition can reverse the hyperglycaemia-induced EMT phenotype [112]. Nonetheless, it was discovered that suppressing FASN was sufficient to trigger EMT and metastasis driven by transforming growth factor beta1 (TGFb1) [113]. These contradictory effects must be investigated further. Another important lipogenic enzyme, acetyl-CoA carboxylase1 (ACC1), is involved in the EMT of breast tumors. It is involved in protein acetylation as well as stimulating by converting acetyl-CoA to malonyl-CoA. According to a recent study, the latter function of ACC1 may control the EMT process. ACC1 inhibition raises acetyl-CoA levels, which leads to the acetylation of Smad2 and the EMT process. Furthermore, leptin or TGF-β signal activation, which is frequent in breast cancer patients with obesity, is linked to lower ACC1 expression, which promotes EMT [114]. As a result, targeting the ACC1-dependent EMT axis could be a promising treatment option for obese breast cancer patients.

A growing body of evidence suggests that a category of enzymes involved in the metabolism of lipids could be used as beneficial targets to stop the EMT development in breast tumors. A major enzyme in arachidonic acid metabolism is a member of the aldo-keto reductase 1 family B1 (AKR1B1) enzyme family that converts prostaglandin H2 to prostaglandin F2a. Twist promotes NF-kB by upregulating AKR1B1 expression through transcription. NF-kB then stimulates Twist expression, resulting in a progressive response loop that activates the EMT program, and improves BCSC-like features. Epalrestat is an anti-AKR1B1 drug that has been shown to drastically reduce EMT. As a result, TNBC may have a therapeutic target in AKR1B1 [115]. Furthermore, the lipid transfer protein (Nir2) acts in place of a unique EMT controller in breast tumor cells. TGFβ1-induced EMT is slowed when Nir2 is silenced, creating Nir a promising beneficial target [116]. Ganglioside 2 (GD2) positive breast tumor cells exhibit BCSC-like characteristics; GD2 has been recognized as a novel indicator for BCSCs [117]. The rate-limiting enzyme for GD2 synthesis- GD3 synthase is used to induce and progress EMT (GD3S). In breast cancerous cells, EMT induces a considerable increase in GD2 concentration and GD3S expression [118]. As a result, blocking GD3S could lead to novel ways to combat breast cancer’s EMT.

Mesenchymal breast cancer cells had higher transcription of genes encoding for triglyceride biosynthesis and lipid droplet production, whereas epithelial breast cancer cells have higher expression of genes involved in de novo fatty acid synthesis, according to proteomic and lipidomic results. As a result, inhibiting triglyceride metabolism could be a viable treatment strategy for preventing EMT [119]. In breast cancer cells, elevated concentrations of phosphatidylcholine and triacylglycerol, and also a reduction in diacylglycerol, were found to accompany the EMT process [120]. More research is needed to fully establish the connection between lipid alteration and the EMT process, and the changes in lipid classes collected in breast tumor cells undertaking EMT. In MCF10A cells, exogenous fatty acids such as linoleic acid as well as arachidonic acid have been found to trigger EMT [121,122]. These findings led to the conclusion that the exogenous fatty acid absorption limitation could be used to investigate EMT induction therapeutically.

### 3.3. Amino Acid Metabolism

Amino acid metabolism influences breast cancer aggression, invasion, and metastasis [123]. According to recent research, numerous important amino acid metabolism enzymes are considerably increased in breast cancer tissues and are linked to high metastatic potential. Moreover, the significance of disordered amino acid metabolism in influencing EMT is not completely established in breast cancer. According to metabolomic studies, the analysis of epithelial and mesenchymal breast cancer cell lines suggests that the mesenchymal phenotype is enriched with more anaplerotic reaction intermediates than the epithelial breast cancer cell line [124]. Thus, indicating the metabolism of amino acids’ significance in the EMT process.

Asparagine is an important amino acid in the EMT process of breast cancer, as evidenced by its high proportion of proteins involved in the EMT program. Moreover, there was a simultaneous reduction in EMT protein expression and low availability of asparagine in breast tumors, as evidenced by elevated levels of aspartate in MCF-7 cell lines compared to healthy breast cell lines. This finding was supported by the low blood levels of aspartate in breast cancer patients, suggesting utilization of aspartate in tumor progression. Such studies advocate that circulating aspartate is an important metabolite feature of breast cancer [125]. The rate-limiting enzyme in asparagine biosynthesis, asparagine synthetase, could be utilized as a therapeutic target to reduce asparagine bioavailability in the tumor microenvironment and could block the EMT program to impair the invasiveness and metastatic potential of breast cancer. Thus, supplementing L-asparaginase or improving the dietary content of asparagine for breast cancer patients prevents the EMT program and reduces metastasis [126,127].

TNBC cells are highly enriched with cystine. On the other hand, luminal subtypes of breast cancer cells are independent of cystine. The result of cystine deprivation induces necrosis in the TNBC phenotype while limiting cell death in the luminal subtype of breast cancer [128]. Transfection of MiR-200c in cystine-enriched breast cancer phenotypes reverses mesenchymal features. Despite the lack of a clear underlying process regarding the involvement of cystine phenotypes of TNBC with the EMT program and the lack of understanding of the underlying processes, the cystine addictive phenotype of TNBC is strongly linked to EMT, which might be a lacuna to execute in detail.

In addition, the progression of TNBC is heavily reliant on glutamine metabolism, which is regulated majorly by the glutaminase isoform [129]. Mainly, glutaminase-2 (GLS2) is involved in boosting mesenchymal markers, invasion, and metastasis [130]. It has been documented that EMT is inversely linked to GLS2 levels. The loss of GLS2 expression during EMT leads to an enhanced glutamine-independent phenotype and decreased mitochondrial activity, while GLS2 restoration in GLS2-negative breast cancer cells exhibits enhanced consumption of mitochondrial glutamine and impairs BCSC-like properties [131]. Further explanations are required for such contradictory findings.

Furthermore, metabolomic analysis is also required in breast cancer patients to monitor the changes in amino acid transporters. Supporting this, an in-vivo analysis of serum metabolites of breast cancer patients showed upregulated inositol-1,4,5-trisphosphate receptor (IPR3) expression in the majority of cases. The lipoproteins and metabolites, namely lactate, lysine, and alanine, were enhanced while serum pyruvate and glucose levels were declined in patients who presented with high IP3R levels compared with healthy individuals [132].

With extensive metabolomics, the glycine biosynthetic pathway had also been highly upregulated in rapidly proliferating breast cancer cells. Thus, glycine supplementation is not recommended in the breast cancer patient’s diet, which might worsen their condition into tumor metastasis and proliferation. It is also suggested that glycine is a potential biomarker and therapeutic response tracker [133].

The GC-MS analysis of breast cancer patients’ serum with subsequent chemometric analysis screens out the changes in metabolite levels. Also, the analysis of metabolic pathways in breast cancer patients mediates enhanced glycolysis, lipogenesis, and the generation of organic metabolites [134]. In support of this, another study compared the metabolic profiling of blood samples of breast cancer patients with healthy women and analyzed 1269 metabolites in different concentrations; among them, 354 metabolites were found associated with proline and arginine metabolism, aminoacyl-tRNA biosynthesis, and bile acid biosynthesis pathways [135]. In addition, stearamide and caproic acid were also significant metabolites linked with breast cancer. Moreover, serum choline, tyrosine, valine, lactate, and isoleucine levels were enhanced, while and glutamate levels were declined in early breast cancer patients. However, advanced metastatic breast cancer patients exhibited declining serum glutamate and glucose levels. In addition, some researchers have claimed that variations in the expression level of oncogenes can be correlated with metabolic profiles, leading to the relapse of breast cancer [135]. Subsequent to this, a study objective was to evaluate serum lipid levels of newly diagnosed invasive breast cancer patients at the I and II stages through NMR profiling of their serum samples. The findings exhibited the linkage of lipoproteins with ER expression in which HDL and Ki67 subfractions were inversely associated. In contrast to this, LDL was found to be linked with the metastasis of breast cancer to lymph nodes. Thus, lipoproteins have been found to be associated with aggressiveness and poor prognosis of breast tumors, suggesting that expression levels of PR and Ki67 can be monitored indirectly [136].

Also, NMR profiling of early or metastatic breast cancer patients showed variations in acetate, acetoacetate, alanine, β-hydroxybutyrate, creatinine, leucine, glucose, glycine, isoleucine lysine, glutamate, glutamine, phenylalanine, pyruvate, threonine, and tyrosine. Specifically, there is an inverse correlation found between tumor size and lactate levels found in early breast cancer tumors, suggesting that tumor cells could modulate a patient’s metabolism at an early stage of breast cancer [137]. Fuss et al. focused on the significance of the overall metabolomic profile besides correlating isolated metabolites due to its greater propensity to predict the prognosis of breast cancer. It was found that choline and taurine were elevated in the intact breast tissue of breast cancer patients compared to benign breast tissue using ex vivo-resolution magic angle spinning (HR-MAS). Also, post-surgery patients (after 5 yrs) were reported to have high creatinine, taurine, and glycerophosphocholine and declined levels of phosphocholine and glycine in malignant tissues [138]. In addition, three significant metabolic clusters were identified from primary tumor samples in HR-MAS analysis in a comparison with untreated breast cancer patients: (1). high level of phosphocholine and glycerophosphocholine; (2). high glucose level; and (3). high alanine and lactate level. Thus, metabolic profiling of breast cancer further validates its heterogeneity [139].

The detailed understanding of metabolic pathways of different breast cancer subtypes could open a new door for discovering potential personalized biomarkers. The advancing heterogeneous classes of breast cancer, especially the glutamine pathway in TNBC, possess an aggressive metabolic pattern. The extensive data quoted here in this manuscript supports the consistent utilization of metabolomics approaches for the establishment of crucial metabolic biomarkers [140].

## 4. Metabolic Inhibitors of EMT Program

Recent research has found that EMT causes considerable metabolic reprogramming. A mesenchyme-specific subgroup with high dihydropyrimidine (DHP) dehydrogenase (DPYD) enzyme-driven synthesis of DHPs was found using unsupervised clustering of metabolism-specific gene expression from a large collection of cancer cell lines [75]. Further research has discovered that chemicals targeted directly at mesenchyme-like cells effectively inhibited glutathione peroxidase 4 (GPX4), a key enzyme in the lipid peroxidase pathway that helps cells avoid ferroptosis, a nonapoptotic form of cell death [141]. EMT-associated transcription factors can directly affect metabolic rewiring (EMT-TFs). For example, ZEB1, a powerful transcriptional EMT regulator, promotes glycosphingolipid metabolism, which enhances the cell’s mesenchymal condition [142]. In breast cancer, SNAI1 controls the inhibition of fructose-1,6-bisphosphatase 1 (FBP1) in order to stimulate glycolysis [90]. As a result, metabolic reprogramming is an unavoidable component of cancer cells’ EMT phenotypic change. Metabolism, on the other hand, has been regarded as an upstream regulator of cellular plasticity. When the TCA cycle enzyme fumarate hydratase (FH) is ablated in renal cancer, fumarate accumulates in the cell, which causes the EMT-suppressing miRNA miR-200 [143] to be epigenetically silenced, allowing EMT-TFs to be activated [144]. Similarly, the nucleotide metabolic enzyme thymidylate synthase (TS), which is generally associated with cell proliferation, has been found to govern the EMT phenotype and breast cancer de-differentiation, requiring DPYD-dependent pyrimidine catabolism [145].

Furthermore, an EMT-focused transcriptome study discovered higher levels of AKR1B1 in mesenchymal cells, indicating that AKR1B1 is involved in EMT and stemness. Excess glucose is converted to fructose in cells via the polyol pathway, a two-step metabolic route driven by aldo-keto-reductase-1 B1 (AKR1B1) and sorbitol dehydrogenase (SORD). The polyol pathway can promote EMT by autocrine TGF-stimulation when engaged by high glucose [146], implying a significant link between glucose metabolism and EMT. An oncometabolite generated by glutamine anaplerosis, D-2-hydroxyglutarate, promoted ZEB1-mediated EMT [147]. Branched-chain amino acid metabolism promotes EMT and metastasis in colorectal cancer through its counterpart enzyme, branched-chain α-ketoacid dehydrogenase kinase (BCKDK) [148]. These findings reveal that metabolic reprogramming and EMT are mutually exclusive phenomena and that EMT may be effectively targeted by priming therapeutic exploitation at the metabolic level. Therefore, we also document some of the metabolic targets and their inhibitors to retard EMT programs (as shown in Table 3) of breast cancer, which reduce cancer stemness, chemoresistance, invasion, migration, immune suppression, and metastatic colonization in distant organs/tissues. In addition, we also detailed some of the repurposed or miscellaneous metabolic inhibitors of the EMT program in breast cancer as tabulated in Table 4.

### EMT-Metabolic Inhibitors at Clinical Levels

EMT transcription and signaling pathways are considered as anticancer drug targets. For EMT transcription pathways: AKT (VQD-OO2 (API-2), KTX-O401 (perifosine), GSK690693, β-catenin (ERX-3722), mTOR (RAD 001 (everolimus)), XL-765 (Exelixis), NF-κβ (OT-304, IMD-0354), PKC (LY317615 (enzastaurin), and for EMT signalling pathways: EGFR-1 (erlorinib, gefitinib), ErbB2 (trastuzumab), IGF-1R (CP-751, 871; AMG479), NOTCH (anti-notch-1 monoclonal antibody), and VEGF/VEGFR (bevacizumab, cediranib) and Src (dasatinib, bosutinib) are under investigation [188,189].

## 5. Conclusions and Future Perspective

In the past decades, there has been a tremendous advancement in molecular targeted therapy with special reference to cancer treatment in the form of precision medicine [190], while the majority of the failures have been reported due to cancer relapse and drug resistance [191]. EMT works to mediate aggressive features of cancer such as cancer stemness, chemoresistance, metastasis, immune suppression, etc., leading to cancer prognosis and is one of the huge hurdles in the development of potential therapeutic interventions against tumorigenesis. In addition, the inhibition of EMT-driven transcription factors through pharmacological agents has been a challenge not yet overcome [192]. However, deep learning of cellular metabolism in different transition states (i.e., epithelial and mesenchymal states) and identification of susceptibilities of each metabolic pathway could provide the possible lacunae for future research and hypothesis to defeat EMT/MET transition in breast cancer [75,144].

In addition, the metabolomic profiling of each breast cancer subtype and the EMT programs associated with them could help us to define certain specific metabolites which can be targeted using potential pharmacological inhibitors/new molecular targets. The therapeutic validation of molecular targets could be attained via detailed exploration of the metabolic rewiring of breast cancer. Finally, the detailed information about the specific metabolic pathways could impact the evaluation of new drugs, with possible repercussions on the survival of breast cancer patients. The prompt identification of chemotherapy-resistant tumors would aid in the earlier and more accurate stratification of patients, and the choice of adjusted therapeutic regimens. The choice of adjusted therapeutic regimens not only involves the targeting of established cytotoxic drugs at the tumor site but also involves the repurposing of metabolic inhibitors of EMT as an adjuvant with the low dose of cytotoxic drugs for breast cancer. Therefore, this review also details some of the metabolic inhibitors of EMT that could be repurposed as an adjuvant with established chemotherapeutic regimens. Moreover, there is ample scope to design selective metabolic inhibitors of the EMT program via in-silico designing for more specific targets, as mentioned in Table 3 and Table 4, to obtain selective outcomes on breast cancer cells for inhibiting metastasis.

## Figures and Tables

**Table 1:** Breast cancer subtypes based on epigenetics.

Molecular Subtype		Normal Breast Like	Luminal A	Luminal B	HER2^+^	Basal Like	Claudin Low
**Hormonal**	**ER**	±	+	±	−	−	−
	**PR**	±	+	±	−	−	−
	**HER2**	−	−	+	+	−	−
**Proliferation Rate**		Low	Low	High	High	High	High
**Frequency of incidence (%)**		5–10	50–60	10–20	15–20	10–20	12–14
**Prognosis**		Intermediate	Good	Intermediate	Poor	Poor	Poor
**P53 mutation**		Low	Low	Intermediate	High	High	High
**Site of metastasis**		Unclear	Bone	Liver, Bone	Lung, Brain	Lung	Unclear

ER-Estrogen receptor; HER2-Human Epidermal Growth Factor Receptor-related protein; PR-Progesterone receptor. Modified from Eroles et al. [18] and Kennecke et al. [19].

**Table 2:** Characteristics and Expression of different EMT markers in breast cancer.

Features/Name of Marker	Phenotype (State of Tissues/Cells)	
	Epithelial	Mesenchymal
**Shape**	Elongated	Rounded
**Motility**	Sessile	Motile
**Adherence**	Adherent to neighbours	Non-adherent to neighbours
**Proliferation**	Higher proliferation	Lower proliferation
**Invasion**	Non-invasive	Invasive
**Micro tentacles**	Absent	Present
	**EMT markers**	
**Expression**	**Epithelial state**	**Mesenchymal state**
**N-cadherin**	Decreased	Increased
**E-cadherin**	Increased	Decreased
**Vimentin**	Decreased	Increased
**Occludin**	Increased	Decreased
**Fibronectin**	Decreased	Increased
**ZO-1**	Increased	Decreased
**MMP-9**	Decreased	Increased
**MMP-2**	Decreased	Increased

**Table 3:** Different metabolites along with metabolic inhibitors to retard EMT program of breast cancer.

S. No.	Metabolite	Metabolite Inhibitors/Metabolic Inhibitor of EMT	Mechanism	Ref.
Glucose Metabolism				
1.	Pyruvate kinase M2 (PKM2)	PKM2 is the limiting step of glycolysis and is strongly related to the EMT process.		[90]
		Fructose 1,6-bisphosphatase (FBP1)	Inhibits PKM2 activation to block glycolysis; boosts mitochondrial complex I activity; enhances OXPHOS; ultimately inhibit EMT.	
		Leptin inhibitors	Leptin promotes EMT via high PKM2 expression and activation of PI3K/AKT signalling cascade. Thus, Leptin receptor inhibition required blocking EMT-induced chemoresistance.	[91]
			LPR-2 inhibits chemotherapeutics resistance on ER^-^ breast cancer cells.	[149]
			Anti-ObR and PI3K/AKT signalling pathway inhibitor LY294002 significantly abolished leptin-induced PKM2 expression and EMT program.	[91]
		Silencing YAP	PKM2 phosphorylation on tyrosine 105 promotes BCSCs via activation of downstream signals of self-associated protein (YAP); enhances nuclear translocation of YAP. Therefore, silencing YAP impairs BCSCs mediated oncogenic kinases and hence inhibit EMT-induced chemoresistance.	[92,93,150]
2.	Pyruvate dehydrogenase kinase 1 (PDK1)	Enzyme prevents pyruvate dehydrogenase complex to initiate TCA cycle; enhance glycolytic metabolism to initiate liver metastases. PDK1 inhibition is necessary for blocking EMT program and prevents liver and lung specific metastasis.		[94]
		Silencing long non-coding RNA- H19	H19 required for glycolytic activity and BCSC characteristics; highly associated with PDK1 expression. H19 silencing abolishes PDK1 expression under hypoxia, glycolysis, and self-renewal circumstances.	[95]
		Aspirin (acetylsalicylic acid)	Inhibits both H19 and PDK1; significantly reduce of BCSC characteristics and block EMT program.	[96]
3.	Phosphoglucose isomerase (PGI)	Catalyzes interconversion of G-6-P and F-6-P; overexpression enhances EMT via increasing NF-kB activity to regulate ZEB transcription.		[97]
		MicroRNA-200	Inhibit ZEB expression and reverses other targets involved in PGI-induced EMT program.	[98]
4.	NADH and NADPH	Significant sources of reducing equivalent ROS detoxification; serve as contributors in decreasing intracellular ROS; NAD (P) H level act as a link between ROS and EMT process. Overexpression of NQO1 promotes PKLR in breast cancer. NQO1 interacts with PKLR to promote glycolysis while preserving NAD (P) H homeostasis.		[103]
		Silencing NQO1	Silencing NQO1 to significantly rise intracellular ROS; which hinders the EMT process.	[104]
5.	Matrix metalloproteinase-3 (MMP-3)	MMP-3 inhibitors	MMP-3 overexpression serves as signal from the breast cancer microenvironment to mediate the ROS in breast cancer cells which further promotes snail and EMT expression.	[107]
**Lipid Metabolism**				
6.	Sterol regulatory element-binding transcription protein 1 (SREBP1)	Main transcriptional promoter of lipogenesis; de novo lipogenesis; inhibit E-cadherin (epithelial marker) expression in breast cancer by forming a co-repressor structure with snail and histone deacetylase.		[110]
		miR-18a-5p	Inhibit SREBP1 to block EMT program and breast cancer lung metastasis.	
7.	Fatty Acid Synthase (FASN)	A lipogenic enzyme required for EMT expansion in breast tumors.		[111,112]
		Cerulenin	Block FASN and slows down the EMT program; also reverses the hyperglycaemia-induced EMT phenotype of breast cancer.	
8.	Acetyl-CoA carboxylase1 (ACC1)	Involved in protein acetylation and stimulates conversion of acetyl-CoA to malonyl-CoA. ACC1 inhibition raises acetyl-CoA level leading to acetylation of Smad2; Leptin or TGF-β signal activation in obese-breast cancer patients to promote EMT program. Thus, targeting ACC1-dependent EMT axis is a promising platform of research in obese breast cancer patients.		[114]
9.	Aldo-keto Reductase 1 family B1 (AKR1B1)	Enzyme converts prostaglandin H2 to prostaglandin F2a. Twist promotes NF-kB activation and induce EMT program to improve BCSC-like features.		[115]
		Epalrestat	Anti-AKR1B1 drug drastically reduces EMT; drug for TNBC targeting AKR1B1.	
10.	Lipid transfer protein (Nir2)	Acts as unique EMT controller in breast tumor cells; TGFβ1-induced EMT is slowed when Nir2 is silenced; thus, it is a promising beneficial target.		[116]
**Amino Acid Metabolism**				
11.	Asparagine synthetase	Rate-limiting enzyme in asparagine biosynthesis and utilized as therapeutic target to reduce asparagine bioavailability in the tumor micro-environment; could block EMT program to impair invasiveness and metastasis of breast cancer.		[126,127]
		L-asparaginase or Asparagine as dietary intake	Supplementing L-asparaginase or improving the dietary content of asparagine for breast cancer patients prevents the EMT program and reduce metastasis.	
12.	Cystine	Cystine deprivation induces necrosis in the TNBC phenotype while limited cell death in the luminal subtype of breast cancer.		[128]
		MiR-200c	Transfection of MiR-200c in cystine-enriched breast cancer phenotypes reverses mesenchymal features.	
13.	Glutaminase-2 (GLS2)	Mediates expression of mesenchymal markers, invasion, and metastasis in TNBC; EMT is inversely linked to GLS2 levels. The loss of GLS2 expression during EMT leads to an enhanced glutamine-independent phenotype and decreased mitochondrial activity, while, GLS2 restoration in GLS2-negative breast cancer cells exhibits enhanced consumption of mitochondrial gluta-mine and impairs BCSC-like properties.		[130,131]
14.	Inositol-1,4,5-trisphosphate receptors (IPR3)	Highly expressed in breast cancer patients with enhanced lactate, lysine, alanine, lipoproteins and low serum pyruvate and glucose levels compared to healthy individuals.		[123]
15.	Glycine	Glycine biosynthetic pathway is highly upregulated in rapid proliferating breast cancer cells. Thus, its supplementation is not recommended in diet which might worsen breast cancer patient’s condition into tumor metastasis and proliferation. Glycine is a potential biomarker and therapeutic response tracking.		[133]
16.	Thymidylate synthase (TS)	Nucleotide metabolic enzyme is associated with cell proliferation, de-differentiation, and EMT phenotype of breast cancer de-differentiation, requiring DPYD-dependent pyrimidine catabolism.		[145]

Abbreviations: anti-ObR: Leptin receptor antibody; F-6-P: fructose-6-phosphate; G-6-P: glucose-6-phosphate; LPR-2: Leptin Receptor peptide antagonist; NQO1: NAD (P) H: quinone oxidoreductase-1; NF-kβ: Nuclear factor-kB; OXPHOS: Oxidative phosphorylation; PKLR- pyruvate kinase expression in the liver and red blood cells.

**Table 4:** Exogenous Blockers of EMT program in breast cancer.

S. No.	Drugs/Formulation	Target	Ref
1.	2-deoxyglucose (2-DG)	Inhibit glycolysis	[151,152]
2.	4-methylumbelliferone	Hyaluronan synthase-2 inhibitor	[153]
3.	Agomelatine	Melatonergic receptors agonist and 5-HT_2C_ antagonist	[154]
4.	Ascorbate	Vitamin C	[155,156]
5.	Apricoxib	COX-2 Inhibitor	[157,158]
6.	Diallyl disulfide	Increases expression of epithelial marker E-cadherin and decreased expression of mesenchymal markers such as Vimentin, N-cadherin and Snail.	[159]
7.	Disulfiram	ALDH1 inhibitor	[160,161]
8.	Epigallocatechin gallate/iron nano-complexes (EIN)	Versatile coating material which eliminates EMT-type cancer cells in-vitro, and in-vivo studies. Thus, inhibit EMT program and improves conventional chemotherapy response via preventing drug chemoresistance.	[162]
9.	Erbulin	A microtubule inhibitor induces MET in TNBC cells and inhibit migration and invasiveness to lungs.	[163]
10.	Etodolac	COX-2 Inhibitor	[164]
11.	L-NAME	pan-NOS inhibitors	[165,166]
12.	L-NMMA	pan-NOS inhibitors	[167,168]
13.	L-tetrahydro-2-furoic acid (L-THFA)	proline dehydrogenase inhibitor	[169,170]
14.	Luteolin	Inhibited cell migration and invasion, and reversed EMT program in dose dependent manner	[171]
15.	Mangiferin	Matrix metalloproteinase (MMP)-7 and -9	[172]
16.	Metformin	AMPK, mTOR inhibitor	[154,173,174]
17.	Olaparib	PARP inhibitor	[175]
18.	Pirfenidone	TGF-β inhibitor	[154]
19.	Propranolol	β-adrenergic receptors antagonist	[154]
20.	Quetiapine	RANK/RANKL inhibitor	[154]
21.	Ribavirin	eiF4E, MNK, IMPDH	[154]
22.	Rifabutin	BCL-6, β-catenin	[154]
23.	Rolipram	PDE4 inhibitor	[176]
24.	Simvastatin	HMG-CoA reductase inhibitor	[177,178,179]
25.	Suramin	Heparinase inhibitor	[180]
**Aptamer**			
26.	EpCAM	Aptamer targeting EpCAM inhibit CSCs linked to siRNAs against PLK1; causes tumor regression when injected in TNBC xenograft model.	[181]
27.	39mer EGFR CL4 aptamer	Impairs the integrin- αvβ3 EGFR complex on TNBC cells	[182]
**MiRNAs**			
28.	miR-10b antagomirs	Inhibit metastasis in a mouse mammary tumor model.	[183]
29.	miR-23a	Its inhibition suppressed the TGF-1-induced EMT, migration, invasion, and metastasis of breast cancer cells	[184]
30.	miR-134	Delivery of miR-134 by exosomes in TNBC cells caused the reduction of cellular migration and invasion.	[185]
31.	miR200c	Expression significantly enhanced the chemosensitivity and decreased the metastatic potential of a p53(null) claudin-low tumor model	[186]
32.	miR520c	Inhibit breast cancer EMT by targeting STAT3 signaling pathway.	[187]

## Data Availability

Not applicable.
